
JOURNAL INFORMATION
==============================
NLM Title Abbreviation: Halls J Health
NLM Journal ID: 0011644

Hall's Journal of Health

Publisher: Biomedical Journal Digitization Project

ARTICLE INFORMATION
==============================
PMCID: PMC9211704
PMID: 36489028
Article version: 1
Subjects: Articles

Health Foods in Hotels

Print publication date: 1879 Aug
Volume: 26
Issue: 8
First page: 31
Last page: 32
License: This work is free of known copyright restrictions. For more information, please see PMC Back Issue Digitization.
License URL: https://creativecommons.org/publicdomain/mark/1.0/

==============================

---

Health Foods in Hotels.—Hundreds ask each month—"What hotels in New York City supply the Health Foods?" To all such we say, that the leading hotel of New York—the Windsor—obtains from the Health Food Co. a variety of its excellent products, takes special care to see that they are properly cooked, and displays them prominently upon its Bills of Fare. The Brevoort—the oldest and best hotel on the European plan in America—cooks the Health Food Company's Gluten, as well as the coarse and fine Granulated Wheats, in the most perfect style, every day in the year. The Sturtevant House placed several of the Health Food Co.'s articles on its Bill of Fare in 1877, and has supplied immense quantities of them to the patrons of this excellent house ever since. Very large quantities of these articles are also made use of by Smith & McNell's Hotel and Restaurant, which feeds 15,000 people daily, the year round. Outside of New York, they are gradually working their way into the best public houses. The Massasoit House, Springfield, Mass., has begun to use them, and the New Haven House, of New Haven, Conn., is consuming them largely. Soon all first-class hotels throughout the country will be forced by public sentiment to serve them regularly. As Mr. Moseley, of the New Haven House says, "No hotel keeper who caters for the best class, can afford to be without them."

---

The Trade Supplied.—The Health Food Co. supply all their products in packages of convenient size. Nearly everything retails at 25 cents per package, and may be obtained of all first-class apothecaries and grocers. Grocers would seem to be the proper persons to keep them, but as they are constantly prescribed by physicians, it is only natural that druggists should seek to monopolize the trade in them. If your Grocer or your Druggist has none of our articles, let him understand that it will pay him to keep them. We append a list of agents from whom supplies may be obtained, should the nearest Druggist or Grocer not have them in stock:

***New York City***—Park & Tilford.
***Brooklyn***—S. B. Rice, 9 Clinton Street.
***Boston***—Fulton, Bartlett & Blain, 604 Washington St. (*Agents for New England*).
***Germantown, Pa.***—Jno. T. Roberts & Bro., 4,934 Main St.
***Buffalo, N. Y.***—Arthur B. Wilkes, 16 East Seneca Street.
***Elizabeth, N. J.***—H. A. Kent, Jr., East Broad Street.
" " Mrs. H. G. Pohle, 221 Broad Street.
***Hartford, Ct.***—Fox & Co.

---

PRINCIPAL OFFICE:

### HEALTH FOOD COMPANY,

***74 Fourth Ave., cor. 10th St.,***

(Adjoining Stewart's). **NEW YORK CITY.**

### HOLLOW SUPPOSITORIES.

We take pleasure in calling the attention of Physicians and Druggists to an invention which supplies what has always been needed—a "*Hollow Suppository*" or *Shell*, made from pure butter of cocoa, which can be kept for any length of time, and can be quickly filled with any medicine required, and prepared for use as promptly as any other prescription. At the same time, it possesses a decided advantage over the ordinary, or solid suppository, from the fact that THE THIN SHELL MELTS AT ONCE ON BEING INSERTED, BRINGING THE MEDICINE IN DIRECT CONTACT WITH THE ABSORBING SURFACES.

The use of Suppositories, as a means of medication in the various diseases of the PELVIC REGION, has wonderfully increased during the past three years. The old plan of treating diseases of the urinary passages and the rectum with medicines taken into the stomach has many disadvantages. Nearly all medicines taken into the stomach have a tendency to fatigue, derange and weaken the digestive organs. Then, the power of the medicine employed is *diffused* throughout the system, and cannot be especially directed to the diseased part, consequently larger and more frequent doses are required to produce the result; and even when a cure is effected it is frequently found that we have only exchanged one disease for another; have, perhaps, produced dyspepsia in the effort to cure constipation by medicines taken into the stomach. Nelaton was of the opinion that CONSTIPATION is a local trouble of the lower bowels, caused by feebleness or inaction of the nerves of the region, or by impacted fecal matter, and that the diseased conditions could be overcome by the use of properly medicated Suppositories, the butter of cocoa dissolving the obstruction, and the medicine allaying the irritation of the mucous surface, strengthening the torpid nerve-ganglia and inducing healthy action. As Suppositories are being prescribed more frequently than ever before, we believe the profession—for the reasons above stated—will generally acknowledge the superiority of the HOLLOW SUPPOSITORIES over all others. They are neatly put up in boxes that contain one gross each, and are sold at about half the price of the solid Suppositories. It costs but a trifle to medicate them as required, and they are fresher, better, cheaper than any other, and far more reliable.

Each box contains printed formulas for the medication of the three sizes, and suggestions for their use, which will be found convenient for reference. The No. 3 Suppositories will be found a useful vehicle in the application of remedies to diseases of the Uterus and Vagina.

### INFANTS AND CHILDREN

are liable to suffer much from the diseases of Constipation. It is all-important to keep the bowels of children regular, so that a healthy condition may be established for life. "*Nelaton's Suppositories*" for *Constipation and all bowel troubles of children*, like "*Nelaton's French Suppositories*," is one of the best of remedies.

### RICORD'S MEDICINAL BOUGIE

is really a *Suppository*, made from butter of cocoa, medicated according to the formula of M. Ricord, of the Venerial Hospital of Paris.

We have found it the easiest, safest and most successful plan of treating specific inflammation of the urethra, whether acute or in the form of *gleet*, generally curing the disease in from two to eight days, and avoiding all danger of *strictures* or inflammation of the *bladder*, and other diseases that so often result from improper treatment.
